# Genomics of Lithium Action and Response

**DOI:** 10.1007/s13311-017-0554-7

**Published:** 2017-06-21

**Authors:** Benjamin S. Pickard

**Affiliations:** Strathclyde Institute of Pharmacy and Biomedical Sciences, University of Strathclyde, 161 Cathedral Street, Glasgow, G4 0RE UK

**Keywords:** Lithium, Bipolar disorder, Pharmacogenomics, Therapeutic response, Toxicity

## Abstract

Lithium is the most successful mood stabilizer treatment for bipolar disorder. However, unlike conventional drugs that are designed to interact with a specific molecular target, the actions of lithium are distributed across many biological processes and pathways. Treatment response is subject to genetic variation between individuals and similar genetic variation may dictate susceptibility to side effects. Transcriptomic, genomic, and cell-model research strategies have all been deployed in the search for the genetic factors and biological systems that mediate the interaction between genetics and the therapeutic actions of lithium. In this review, recent findings from genome-wide studies and patient cell lines will be summarized and discussed from a standpoint that genuine progress is being made to define clinically useful mechanisms of this treatment, to place it in the context of bipolar disorder pathology, and to move towards a time when the prescription of lithium is targeted to those individuals who will derive the greatest benefit.

**Electronic supplementary material:**

The online version of this article (doi:10.1007/s13311-017-0554-7) contains supplementary material, which is available to authorized users.

## Introduction to Lithium, the Mood Stabilizer

The serendipitous discoveries of therapeutic drugs are a fascinating chapter in twentieth-century medicine. The application of lithium as a treatment for mood disorders is one of the key examples. In the late 1940s, John Cade, a psychiatrist working in Melbourne, Australia accidentally discovered lithium’s sedative properties during rodent experiments designed to explore the role of uric acid in psychiatric conditions (although Cade knew about lithium as a historical treatment for uric acid build up in gout) [1]. After a lengthy lag period caused by concerns over its safety, the clinical development of lithium for “psychotic excitement”, as Cade originally termed it, accelerated in the 1960s and 1970s. Fifty years on, lithium is still the “gold standard” treatment for bipolar disorder with cumulative and overwhelming evidence for its therapeutic effectiveness and consequent life-saving outcomes [2, 3].

In an era of rational high-throughput drug screening of complex libraries chosen to represent the full dimensionality of chemical space, it is somewhat bemusing to discover that the blunt tool of a metal ion is a top-performing mood stabilizer. This is a “small molecule” but without a single defined “target”. Lithium ions possess a single positive charge and have a radius of 76 pm [4]. We must assume that these 2 properties alone mediate its pharmacological action, most likely by mimicking and compromising the actions and targets of more physiologically relevant trace metals with similar physical properties such as Cu^+^ (77 pm), Mg^2+^ (72 pm), Zn^2+^ (74 pm), and the “high spin” form of Fe^2+^ (78 pm).

There is a stock view presented in numerous reviews of therapeutic lithium that we understand little about the biological mechanisms of its action. The sheer scale of lithium’s potential physical interactions perhaps explains why it has been so difficult to define its major biological modes of action—both good and bad—until the advent of more sophisticated and objective methodologies. A second confounding factor has been the relative invisibility of lithium atoms in X-ray crystallography. Consequently, any presumed functional interaction with a protein, such as glycogen synthase kinase 3β (GSK3β), has been very hard to confirm or characterize. The physical association of lithium and magnesium ions with the phosphate chain of energy intermediate molecule, adenosine triphosphate, has only recently been inferred by nuclear magnetic resonance, and demonstrated to alter its purinergic signaling capacity [5]. Likewise, lithium’s pharmacological action within the catalytic site of (bacterial) inositol monophosphatase has only been indirectly observed through magnesium displacement upon increasing lithium concentrations [6]. This review will acknowledge the technical challenges facing the field, but will also offer encouraging evidence for progress from recent studies of the mechanisms of lithium’s therapeutic action, the basis for its side effects, and the genetic determinants of patient response.

## Side Effects of Lithium

Given the lack of specificity, it is surprising that lithium’s therapeutic action is so potent and the toxic side effects not more pronounced. It is, however, a close-run race—the therapeutic index of lithium is very narrow, indicating dosages with therapeutic action are close to dosages with toxic side effects. Patients undergoing lithium treatment require regular blood tests to ensure that safe and effective levels are maintained. They also undergo occasional assessment of kidney and parathyroid function: the 2 organs most susceptible to lithium toxicity. This potential for harm provides a strong motivation to deconvolute the biological action of lithium to identify targets that could be pharmacologically manipulated with more specific and less risky drugs and, in addition, to define harmful “off-target” effects.

Multiple side effects of lithium have been described since it was licensed for the treatment of acute mania and then bipolar disorder in the 1970s. These include hypothyroidism, nausea, diarrhea, and weight gain. However, it is the damage to the kidneys correlated with long-term use and excessive serum concentrations that is the most well documented and characterized [7–9]. This primarily manifests as a common (~50% of patients) increase in thirst resulting from polyuria—excessive urination—and, less commonly, as a progression to nephrogenic diabetes [10]. Both states indicate a failure to concentrate urine in the collecting duct of the nephron. The epithelial cells of the collecting duct permit the entry of lithium through amiloride-sensitive ENaC sodium channels. As an aside, a recent paper described a screen for single-nucleotide polymorphism (SNP) variants in selected candidate genes that predispose those prescribed with lithium to long-term deterioration of estimated glomerular filtration rates [11]. A CC genotype at SNP rs378448, located in *ACCN1*, was identified as the most significant predisposing factor. Additionally, a SNP in the first exon of this gene has been associated with lithium response in a small Sardinian cohort [12]. *ACCN1* encodes a member of the ENaC superfamily, but it is not known if this plays a role in the collecting duct pathology. Once lithium enters the duct cells, there are many and, in some instances, hotly debated routes of pathological action, including increased cyclooxygenase-2 expression generating prostaglandin E_2_, inhibition of GSK3β/vasopressin action, decreased urea transporter expression, increased cell-cycle activity leading to cellular remodeling, and reduced inositol monophosphate/cyclic adenosine monophosphate signaling [13–15]. Ultimately, there is reduced induction and expression of the aquaporin-2 channels in the duct cell wall, preventing the passive reuptake of water from the duct and therefore leading to the production of excess, dilute urine. Much less common (~1%) in those prescribed lithium is a further chronic progression to nephrotoxicity and histologically visible damage to the kidney [16].

Adding to the complexity of the dosage balancing act is the variable lithium response exhibited by each patient. Approximately 30% of those prescribed lithium exhibit a good response, with the remainder falling into partial- and nonresponder groupings. This spectrum may be owing to heterogeneous genetic and environmental components—certainly there is good evidence for heritable response to lithium in bipolar disorder family clusters [17, 18]. Other factors that correlate with response have been comprehensively reviewed previously and include age–at–onset and periods of remission [19]. Patient response might also directly relate to genetically determined variation in lithium’s physiological availability, excretion and molecular targets. For example, in the rat, the major determinant of lithium reabsorption back into the bloodstream from the kidney glomerular filtrate was recently identified as a foscarnet- and parathyroid hormone-sensitive sodium–phosphate co-transporter activity. This raises the possibility that human genetic variation at this locus/loci could significantly influence the half-life of circulating lithium in the bloodstream [20].

## Archetypal Mediators of Lithium’s Therapeutic Effects

In terms of lithium’s therapeutic action, there are a number of classically studied proteins and pathways that are definitive lithium targets. These have been reviewed extensively elsewhere [21, 22]; only 2 will be briefly summarized here. First is lithium’s known inhibition of the protein GSK3β [23–25]. This protein is a component of the WNT signaling pathway, inhibition of which allows the accumulation of β-catenin protein and consequent downstream gene regulation. GSK3β also regulates other proteins (e.g., arrestins) and is itself regulated by other pathways (e.g., Akt). Second, an excess of inositol is believed to be a biochemical pathology in bipolar disorder affecting multiple cellular systems, including mitochondrial function, autophagy, growth cone function, and calcium signaling. Lithium depletes this excess signaling by directly inhibiting inositol monophosphatase 1—diverting the metabolic pathway that generates inositol.

## Transcriptomic Approaches Struggle to Reveal Lithium Action

In addition to these established modes of action, there are now multidisciplinary approaches to fully characterize lithium’s biology and the factors that influence a patient’s response. One approach is to employ transcriptomic methods to identify genes up- or downregulated in response to lithium treatment. The premise is that these expression changes will reflect the principal target genes and biological pathways that underlie therapeutic action. However, the results have been disappointingly inconsistent. For this review, an informal analysis was carried out on differentially expressed gene sets from 5 representative transcriptomics papers spanning human/rodent studies and *in vitro*/*vivo* sample types [26–30]. This identified only 2 genes, *CD93* and *SULT1A1*, shared between any 2 studies. A more sophisticated analysis of this kind was carried out by Breen *et al*. [31], to act as validation of their own gene expression study. The authors identified a number of results shared across publications, including *STC2*, *HADH*, *GAMT*, *MAT2A*, *HSP90AA1*, *CRIP1*, *CKB*, *FOS*, *LAX1*, and *RSAD2*, but, further indicating the difficulties of the gene expression approach, these genes were not identified in the studies assessed in this review. Many of these inconsistencies may lie in the choice of cell type, treatment regimen, and even species.

## Induced Pluripotent Stem Cells Offer a Means to Explore Lithium Response in Well-Controlled Human Models

The emergence of induced pluripotent stem (iPS) cell technologies has permitted the detailed investigation of mental illness in biologically relevant human cell types. The acute nature of the brain’s response to lithium has been convincingly modeled in an *in vitro* study of dentate gyrus-like neurons derived from 6 patients with bipolar disorder and 4 healthy control iPS cell lines [32]. Disease-associated phenotype differences observed in the patient neurons included altered mitochondrial function, abnormal calcium signaling, and, most noteworthy, a general hyperexcitability manifested as increased sodium ion currents and increased frequency of spontaneous action potentials. The authors selected 3 good lithium responders and 3 nonresponders when recruiting the cohort with bipolar disorder. The hyperexcitability phenotype was reversed by lithium treatment in the good responder subset alone: extraordinary evidence that the heterogeneous nature of complex genetic phenotypes can occasionally “collapse” into a simple and discriminating marker. The authors went on to identified 45 genes with altered expression in bipolar patient lines and biological categorization of these confirmed the involvement of mitochondrial processes. Importantly, the number of genes with significant expression changes in response to lithium treatment was an order of magnitude greater in the responding cells than in the nonresponding cells. Among these genes were a number that have been previously implicated in bipolar disorder (*PDE11A*, *PRKCH*, *PTPRB*, *SCN11A*, *NKAIN*, *KCNA1*, and *KCNJ12*), indicating that a good response to lithium correlates with a partial rescue of pathological gene expression profiles. One of the disease-associated, lithium-responding genes, *KCNA1*, was also identified in the study by McQuillin *et al*. [26]. This gene encodes a potassium channel, proposed to mediate a homeostatic response to the cellular hyperexcitability: expression increases in disease, but decreases as lithium reverses this electrophysiological phenotype. Similarly, the disease-associated, lithium-responding gene *Pde11a*, encoding a phosphodiesterase that catabolizes cyclic adenosine monophosphate and cyclic guanosine monophosphate, was shown to be expressed at a lower levels in the hippocampi of mouse strains that respond to lithium [33]. *Pde11a* knockout mouse strains also displayed an increased response to lithium compared with wild-type littermates.

The iPS work above has been neatly complemented by a recent publication that used lymphoblastoid cell-derived iPS cells from patients with bipolar disorder and healthy control cohorts of the same size and composition as above [34]. When differentiated into dentate gyrus-like granule cell neurons, those from patients with bipolar disorder also displayed an increased likelihood of the hyperexcitable phenotype. However, a more discerning electrophysiological characterization of the cells revealed that lithium responder and nonresponder lines achieved this hyperexcitability through different perturbations in their underlying sodium and potassium channel activity and action potential parameters, with distinct electrophysiological changes after lithium treatment. This enabled the construction of a lithium response prediction model based on electrophysiology alone.

A third study employing iPS cells generated from responsive and nonresponsive patients focused on the proteomic changes that follow lithium treatment [35]. Of the 15 proteins identified with altered expression or gel migration, collapsin response mediator protein 2 (CRMP2) was chosen as an attractive candidate for further study as it showed increased phosphorylation on threonine position 514 in lithium-responsive patient iPS-derived neurons. This site can be phosphorylated by GSK3β (among other kinases), and both lithium and a specific GSK3β inhibitor were shown to reduce its phosphorylation level. CRMP2 regulates the cellular response to semaphorin 3A (formerly known as “collapsin”), an extracellular signaling molecule that binds to neuropilin/plexin receptors. This pathway shapes dendritic spines and axonal growth cone morphology during development and in the adult—with implications for synapse formation and function. Spine morphologies were shown to be altered *in vitro* and in postmortem brain samples, suggesting this is a genuine bipolar disorder pathology, and one that can be ameliorated by lithium, most likely through action on the GSK3β/CRMP2/semaphorin 3A pathway. The antidepressant tianeptine, antiepileptic lacosamide, and neurotrophic brain metabolite lanthionine ketimine have all been shown to act on CRMP2 [36].

Together, these studies have created overlapping frameworks of disease pathology and drug action that significantly advance our understanding and provide discerning functional assays for drug development and pharmacogenomic profiling.

## The Era of Genome-Wide Studies Into Lithium Response

Objective genome-wide approaches to study complex genetic disorders, such as SNP-based association and high-throughput sequencing, have also been instrumental in the search for novel facets of pathology and pharmacogenomics across the full range of complex genetic disorders. A key step in the assessment of disease-predisposing genetic risk factors in association studies is to compensate for false positives that will inevitably arise when genotyping hundreds of thousands of genetic variants in each individual—patient or healthy control. Rather than use the standard statistically significant *p*-value threshold of 0.05, the requirement is for a *p*-value of 10^–8^ to be met, or surpassed, before a finding can be considered “genome-wide significant”. For bipolar disorder, the emergence of genome-wide significant variants within calcium channel genes perhaps offers the most pharmacologically tractable finding from the multitude of genes discovered and validated [37, 38]. Direct assessment of lithium response factors via genome-wide association study has been reported in 4 studies with sufficient participants to be considered adequately powered (see Table 1 for summary). The phenotypic definition of lithium response at the level of the patient is most often achieved using the ‘Alda’ rating scale based on case notes and prescription history [17]. As with all psychiatric disorders, there is no other clear physical biomarker that can be employed for this purpose. Discounting the new evidence for *in vitro* electrophysiological changes, there is only one *in vivo* physical measure that could act as a quantifiable phenotype: the robust association of brain region size increases that correlate with extended lithium use [39]. A correlation with lithium response has not been tested (although perhaps inferable from long-term use) but would be an indication that cell proliferation/cell size are additional pathologies worthy of exploration. The first of the 4 genome-wide studies failed to reach statistical significance at any SNP locus [40]. However, the second study, despite being made up of only 264 patients with bipolar disorder, and then a further 100 patients in a validation study, achieved extremely significant *p*-values for SNP markers within *GADL1* [41]. Depending on the parameters used in the definition of lithium response, the *p*-values for the most significant SNP, rs17026688, were between 9.19 × 10^−18^ and 5.50 × 10^−37^. This extraordinary level of significance, and associated effect size, should be practically predictive for patient lithium response. The gene encodes the glutamate decarboxylase-like protein 1 that, because of its sequence similarity to γ-aminobutryic acid-ergic metabolism enzymes glutamate decarboxylase 1 and 2, appears a plausible candidate. However, *GADL1* is seemingly not expressed in the brain and is principally engaged in taurine metabolism [42]. While there is validation of this GWAS finding in a second study in the Han Chinese population [43], other population studies have failed to replicate association [44, 45], which suggests that the gene’s candidacy should be treated with a level of caution until a statistical consensus emerges. In the third study, a number of association comparisons were made across several collected cohorts in which lithium response was either subjectively (self-) reported or objectively assessed [46, 47]. The only case–control permutation which yielded genome-wide statistical significance was between 387 objectively assessed lithium responders and 6684 healthy controls. This pinpointed a SNP, rs146727601 (*p*-value:1.22 × 10^−9^), located within the gene, *PTS*, encoding 6-pyruvoyltetrahydropterin synthase, an enzyme that participates in the synthesis of the cofactor tetrahydrobiopterin (BH_4_). BH_4_ aids the production of nitric oxide and monoamine neurotransmitter synthesis. However, a mouse knockout of *Pts* alters metabolism and abdominal fat distribution without an overt central nervous system phenotype or perturbation of neurotransmitter levels [48]. There are other SNPs in this study that fall marginally below the genome-wide statistical threshold but which have intriguing links to established psychiatric biology, suggesting that larger surveys are required. Finally, a fourth study carried out by the Consortium on Lithium Genetics (ConLiGen) consisted of a genome-wide association study of 2563 individuals diagnosed with bipolar disorder and phenotypically assessed for lithium response using both continuous and categorical assessment tools [49]. Marginally sub-genome-wide significant SNPs were identified using the continuous phenotypic model in the genes *CSMD2* [rs9662615 (*p* = 5·26×10^–7^); gene previously implicated in multiple neuropsychiatric diagnoses [50]] and *HDAC9* [rs61549860 (*p* = 5·44×10^–7^); gene disrupted in one reported case of schizophrenia [51]]. However, a cluster of genome-wide significant SNPs was identified on chromosome 21 over a potential long noncoding mRNA associated with an expressed sequence tag with accession number AL157359 (most significant SNP rs74795342; *p* = 3·31×10^–9^). The biological significance of this transcript is currently unknown.

**Table 1:** Summary of the top findings from 3 genome-wide association studies of lithium response

SNP identifier	Chromosome	Implicated gene	*p*-value	Comment
Chen et al. [41]				
rs17026688	3	*GADL1*	5.50 × 10^−37^	Discovery cohort
Hou et al. [49]				
rs79663003	21	*AL157359*	1·37 × 10^–8^	European/Asian ancestry cohorts combined
rs78015114	21	*AL157359*	1·31 × 10^–8^	European/Asian ancestry cohorts combined
rs74795342	21	*AL157359*	3·31 × 10^–9^	European/Asian ancestry cohorts combined
rs75222709	21	*AL157359*	3·50 × 10^–9^	European/Asian ancestry cohorts combined
rs9662615	1	*CSMD2*	5·26 × 10^–7^	European cohort alone
rs771148	1	*CSMD2*	7·01 × 10^–7^	European cohort alone
rs61549860	7	*HDAC9*	5·44 × 10^–7^	European cohort alone
Song et al. [46, 47]				
rs146727601	11	*PTS*,<space> *PLET1*	1.22 × 10^−9^	Corrigenda *p*-value
rs77866734	19	*TCF3*, *KIR3DP1*, *KIR2DL4*	7.59 × 10^−7^	Corrigenda *p*-value

*p*-Values in bold indicate genome-wide significant findings

## Conclusions

It is evident that research findings in the last few years have sharpened our resolution of the processes that are affected by lithium treatment in the context of bipolar pathology. Results from differentiated iPS cells have been the most perceptive—revealing disorder-associated protein and electrophysiological changes that are reversed by lithium and distinguish patient response profiles. Such cells will be vital tools to help bridge the biological gap between the known genetic factors controlling lithium response and quantifiable electrophysiology. With current advances in microfluidic devices that model neural networks and the emergence of organoid production, we may soon be able to examine lithium’s effects at higher functional levels: brain structural changes and the connectivity changes that might underlie its mood stabilizing activities. Genomic studies, however, are still on a cusp—mirroring the state of complex disease gene identification some 5 years ago, immediately before meta-analyses pushed case numbers into the high thousands and unleashed a torrent of significant SNP findings. Doubtless that will come, and in the meantime we should be cautious not to over-interpret. Ultimately, the hope is that the genomic Venn diagram of lithium response and bipolar disorder risk will overlap in a restricted but insightful manner and, in this way, give drug companies clear direction for the development of equally potent but safer alternatives to lithium.

## Electronic supplementary material

Below is the link to the electronic supplementary material.

ESM 1 Required Author Forms Disclosure forms provided by the authors are available with the online version of this article. (PDF 1647 kb)
